# The Health Effects of Climate Change: A Survey of Recent Quantitative Research

**DOI:** 10.3390/ijerph9051523

**Published:** 2012-04-25

**Authors:** Margherita Grasso, Matteo Manera, Aline Chiabai, Anil Markandya

**Affiliations:** 1 Budgeting and Planning, Enel Engineering and Innovation, S.p.A., 20121 Milan, Italy; Email: margheritastellagrasso@gmail.com; 2 Department of Statistics, University of Milan-Bicocca, 20126 Milan, Italy; 3 Fondazione Eni Enrico Mattei, 20136 Milan, Italy; 4 BC3 Basque Centre for Climate Change, E-48008 Bilbao, Spain; Email: aline.chiabai@bc3research.org (A.C.); anil.markandya@bc3research.org (A.M.); 5 Department of Economics, University of Bath, BA2 7AY Bath, UK

**Keywords:** climate change, health, quantitative models, non-infectious diseases, infectious diseases, malaria, cardiovascular diseases

## Abstract

In recent years there has been a large scientific and public debate on climate change and its direct as well as indirect effects on human health. In particular, a large amount of research on the effects of climate changes on human health has addressed two fundamental questions. First, can historical data be of some help in revealing how short-run or long-run climate variations affect the occurrence of infectious diseases? Second, is it possible to build more accurate quantitative models which are capable of predicting the future effects of different climate conditions on the transmissibility of particularly dangerous infectious diseases? The primary goal of this paper is to review the most relevant contributions which have directly tackled those questions, both with respect to the effects of climate changes on the diffusion of non-infectious and infectious diseases, with malaria as a case study. Specific attention will be drawn on the methodological aspects of each study, which will be classified according to the type of quantitative model considered, namely time series models, panel data and spatial models, and non-statistical approaches. Since many different disciplines and approaches are involved, a broader view is necessary in order to provide a better understanding of the interactions between climate and health. In this respect, our paper also presents a critical summary of the recent literature related to more general aspects of the impacts of climate changes on human health, such as: the economics of climate change; how to manage the health effects of climate change; the establishment of Early Warning Systems for infectious diseases.

**JEL Classification:** C2; C3; I1; Q54

## 1. Introduction: Some Facts and Opinions on the Relationship Between Climate Change and Health

In recent years there has been a large scientific and public debate on climate change and its direct as well as indirect effects on human health. According to [1], some 2.5 million people die every year from non-infectious diseases directly attributable to environmental factors such as air pollution, extreme weather events, stressful conditions in the workplace, exposure to chemicals such as lead, and exposure to environmental tobacco smoke.

In particular, lead exposure has been estimated to account for 2% of the ischaemic heart disease burden and 3% of the cerebrovascular disease burden [2]. Exposure to outdoor air pollution accounted for approximately 2% of the global cardiopulmonary disease burden [2]. In the U.S., about 12% of the ischaemic heart disease burden has been related to occupation, for the age group 20–69 years. This estimate has been based on the specific risk factors of job control, noise, shift work and environmental tobacco smoke at work [3]. In Finland, it has been estimated that occupational risks account for 17% of the deaths from ischaemic heart disease, and 11% of those from stroke [4]. In Denmark, the occurrence of cardiovascular diseases is related to the type of occupation. Specifically, a reduction of 16% (22%) in the cardiovascular disease burden can be attributable to men (women) with non-sedentary occupations [5]. Changes in climatic conditions and climate variability represent a further factor which can affect human health directly or indirectly via changes in biological and ecological processes that influence the transmission of several infectious diseases [2]. Direct effects on human health include, for example, thermal stresses due to increased frequency and intensity heat waves (cardiovascular and respiratory diseases, heat exhaustion), and deaths and injuries due to extreme weather events. Indirect effects include malnutrition, food-, water- and vector-borne diseases, together with increased morbidity due to the combined effect of exposure to high temperature and air pollution.

Empirical evidence suggests that malaria varies seasonally in highly endemic areas and is probably the vector-borne disease more sensitive to long-run climate changes. For example, the comparison of monthly climate and malaria data in highland Kakamega, Western Kenya, highlights a close association between malaria transmission and monthly maximum temperature anomalies over the years 1997–2000 [6]. The effects of soil moisture to determine the causal links between weather and malaria transmission has been studied by [7]. For the most common mosquito species *Anopheles gambiae*, the soil moisture predicts up to 45% and 56% of the variability of human biting rate and entomological inoculation rate, respectively. The link between malaria and extreme climatic events has long been the subject of study on the Indian subcontinent as well as in various other countries. Early in the twentieth century, the Punjab region experienced periodic epidemics of malaria. Excessive monsoon rainfall and the resultant high humidity were clearly identified as major factors in the occurrence of malaria epidemics. More recently, time-series analyses have shown that the risk of a malaria epidemic increased approximately five-fold during the year following an El Niño event in the Indian region [8]. Furthermore, a strong correlation is found between both annual rainfall and the number of rainy days and the incidence of malaria in most districts of Rajasthan and in some districts in Gujarat [9]. The relationship between reported malaria cases and El Niño has also been documented for Venezuela, where, during the whole twentieth century, malaria rates increased on average by over one-third in the year immediately following an El Niño event [10].

However, it is widely acknowledged that climate changes are only one of many important factors influencing the incidence of infectious diseases and that their effects are very unlikely to be independent of socio-demographic factors (e.g., human migrations, transportation, nutrition), or of environmental influences (e.g., deforestation, agricultural development, water projects, urbanization). In particular, it has been estimated that about 42% of the global malaria burden, or half a million deaths annually, could be prevented by environmental management, although this proportion varies significantly across different regions: it is 36% in the Eastern Mediterranean Region; 40% in the Western Pacific Region; 42% in sub-Saharan Africa; 42% in the South-East Asia Region; 50% in the European Region; 64% in the Region of the Americas [1].

Nevertheless, in the past fifteen years a large amount of research on the effects of climate change on human health has addressed two fundamental questions [2]. First, can historical data be of some help in revealing how short-run or long-run climate variations affect the occurrence of infectious diseases? Second, is it possible to build more accurate statistical models which are capable to predict the future effects of different climate conditions on the transmissibility of particularly dangerous infectious diseases? The primary goal of this work is to review the most relevant contributions which have directly tackled those questions, with respect to the effects of climate changes on the diffusion of non-infectious and infectious diseases. Specific attention will be drawn on the methodological aspects of each study, which will be classified, according to the type of quantitative model considered, in the following categories [11]:

- **Time series models**, among which ARMAX (Auto Regressive Moving Average with exogenous variables) models, ECM (Error Correction Models), and non-parametric forecasting models such as single and double exponential smoothing, Holt-Winters methods (additive, no seasonal, multiplicative);
- **Panel data and spatial models**, such as fixed and random effects models, dynamic panel data models, spatial lag and spatial error models;
- **Non-statistical approaches**, such as Integrated Assessment Models (IAMs), Computable General Equilibrium (CGE) models, Global Trade Analysis Project Models (GTAP), and Comparative Risk Assessments (CRA).

Since many different disciplines and approaches are involved, a broader view is necessary, in order to provide a better understanding of the interactions between climate and health. In this respect, our paper also presents a critical summary of the recent literature related to more general aspects of the impacts of climate changes on human health, such as: the economics of climate change; how to manage the health effects of climate change; Early Warning Systems and infectious diseases; evaluating the risks to human health which are related to climate change; the implications of modifiable environmental risk factors.

The paper is organized as follows: Section 2 presents the most popular classes of quantitative models used to analyze the relationship between climate variations and the diffusion of non-infectious and infectious diseases. In particular this section briefly illustrates the different methodologies and discusses, for each approach, several examples from the relevant empirical literature. Section 3 integrates the results from multiple quantitative studies which are focused on malaria. Section 4 presents a critical summary of the recent literature related to the following more general aspects of the impacts of climate changes on human health. Section 5 concludes. 

## 2. Quantitative Models for the Relationship Between Climate Change and Health: Methods and Examples

Quantitative models are important tools for analysing the complex relationship between climate changes and human health, since they allow researchers to link crucial climate variables (such as temperature and precipitations) at global or regional levels to the occurrence of the disease under scrutiny [2]. In this section, we briefly describe each approach, and for each approach we discuss several examples from the relevant empirical literature. 

### 2.1. Time Series Models

#### 2.1.1. Methods

In applied statistics, the standard model that takes into account the random nature and time correlations of the variable under study (e.g., the occurrence of a particular disease), *Y_t_*, *t* = 1,…,*T*, is the Auto Regressive Moving Average model, where the autoregressive component is of order *p* and the moving average part is of order *q*, or ARMA(*p*, *q*) (see, among others, [12]): 


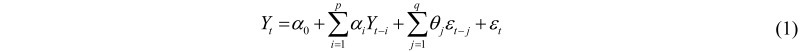


In order to describe the relationship between the occurrence of a specific disease and climatic variables more accurately, an Auto Regressive Moving Average model with exogenous variables can be used. The notation ARMAX(*p*,*q*,*b*) refers to a model with *p* autoregressive terms, *q* moving average terms and *b* exogenous variables:


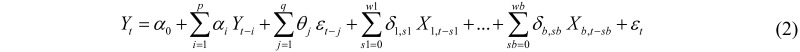


A number of variations of ARMA models are commonly used in statistics, according to whether the series *Y_t_* and *X_r,t_* are non-stationary, given the presence of deterministic and/or stochastic trends, or exhibit seasonalities.

The relationship between two non-stationary variables *Y_t_* and *X_t_*, both integrated and cointegrated, can be represented via an Error Correction Model (ECM), with possible asymmetric terms:


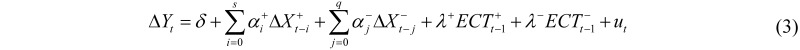


where Δ*X_t_* = *X_t_*−*X_t−_*_1_; Δ*X*^+^ = Δ*X* if Δ*X* ≥ 0 and Δ*X*^+^ = 0 otherwise; Δ*X*^−^ = Δ*X* if Δ*X* < 0 and Δ*X*^−^ = 0 otherwise; *ECT_t_* are the residuals from the cointegrating regression of *Y_t_* on *X_t_*; *ECT*^+^ = *ECT* if *ECT* ≥ 0 and *ECT*^+^ = 0 otherwise; *ECT*^−^ = *ECT* if *ECT* < 0 and *ECT*^−^ = 0 otherwise. Parameters *α*^+^ and *α*^−^ are the short-run marginal effects, while parameters *λ*^+^ and *λ*^−^ are the speeds of adjustment of *Y_t_* from *t−*1 to *t* to the equilibrium, once a disequilibrium has occurred in *t−*1.

Many economic, socio-demographic, environmental and climatic variables exhibit seasonal behaviour. As in the case of trends, the time series literature distinguishes between deterministic and stochastic seasonality. A non-stationary time series *Y_t_*, observed at *S* equally spaced time intervals per year, is said to be seasonally integrated of order *d*, or SI(*d*), if Δ*_S_^d^Y_t_* is a stationary and invertible ARMA process of the type described by equations (3) [13]. The simplest seasonal model for non-stationary variables is the seasonal random walk (SRW): *Y_t_* = *Y_t-S_* + *ε_t_*. The SRW model can be generalized to the seasonal integrated ARMA (SARIMA) model:


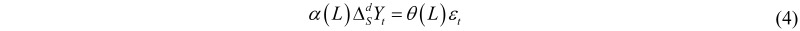


where the AR polynomial *α*(*L*) and the MA polynomial *θ*(*L*) in the lag operator *L* have all roots outside the unit circle, *i.e.*, the AR part of equation (4) is stationary, while the MA part of equation (4) is invertible.

Exponential smoothing is a method of adaptive forecasting, which is useful in cases where the number of observations on which to base the forecasts is limited. The basic idea underlying exponential smoothing is that forecasts adjust on the basis of past forecast errors [14]. If *Y_t_*, *t* = 1,..., *T*, is the time series to be predicted and Y_t_^*^ is the smoothed series, Y_t_^*^ is calculated according to the following recursive model:


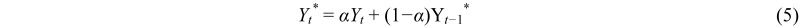


where 0 < α ≤ 1 is the smoothing factor. The smaller is α, the smoother is *Y_t_*. Model (5) is referred to as single smoothing, and is appropriate for stationary, non-seasonal time series. By repeated substitutions in (5), Y_t_^*^ can be written as a weighted average of past values of *Y_t_*, where the weights (1*−α*)*^t^* decline exponentially with time. The out-of-sample forecasts from single smoothing are constant for all observations and are given by: Y_T+h_^*^ = *Y_T_*, for all *h* > 0, *h* = *T* + 1,…,*T* + *H*. The method known as double smoothing applies single smoothing twice and is appropriate for time series which are non-stationary for the presence of a linear deterministic trend.

A method which is suitable for a time series with a linear trend and additive seasonal variations is the so-called additive Holt-Winters. The smoothed series is given by:


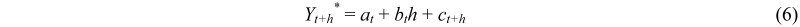


where *a_t_* and *b_t_* are the permanent component and trend parameters, while *c_T+h_* represent the additive seasonal factors.

If *Y_t_* is a time series characterized by the presence of a linear trend and multiplicative seasonal variability, the multiplicative Holt-Winters model is typically applied. In this case, the smoothed series is given by the following modified version of (6):


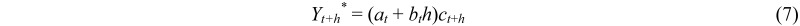


#### 2.1.2. Examples

Time series models have been used extensively for predicting the evolution pattern of diseases, and more specifically to assess the relationship between environmental exposure and mortality or morbidity over long time periods. [15] found that the dynamics of the cutaneous leishmaniasis are strongly associated with climate indicators, such as temperature and ENSO, and that linear models can provide satisfactory forecasting of the incidence of the disease. These predictions are a necessary step for quantifying the potential impact of climate on health and the related costs. In the field of climate based Early Warning Systems (EWS), which are used to predict the occurrence of epidemics of infectious diseases, [16] review and compare linear and non-linear models for forecasting seasonal time series of diseases. Using American cutaneous leishmaniasis, as an example, the models are evaluated based on the predictive R^2^ for forecasting the data “out-of-fit”. Seasonal autoregressive models that incorporate climatic covariates are found to provide the best forecasting performance. Additionally, a simulation exercise shows that the relationship of the disease time series with the climatic covariates is strong and consistent for the seasonal autoregressive (SAR) modeling approach. While the autoregressive part of the model is not significant, the exogenous forcing due to climate is always statistically significant. Prediction accuracy can vary from 50% to over 80% for diseases burdens at time scales of one year or shorter. Other time series studies in the context of cutaneous leishmaniasis include [17,18]. A different strategy for predicting the pattern of diseases is given by [19], who investigate the dynamics of diarrhea, acute respiratory infection (ARI), and malaria in Niono, Mali. The authors observe that these disease time-series often *(i)* suffer from non-stationarity; *(ii)* exhibit large inter-annual plus seasonal fluctuations; *(iii)* require disease-specific tailoring of forecasting methods. To accommodate these characteristics they suggest using a non-parametric technique, the multiplicative Holt-Winters method (MHW). This is a recursive method that can be described as follows: *(i)* based on past information and pseudo-parameters initialization the MHW produces point forecasts (the method also decompose the time series into level, trend (rate of change), seasonal, and approximately serially uncorrelated residual TS components); *(ii)* point forecasts are recursively revised through residuals bootstrap to produce median forecasts and their 95% confidence interval bounds; *(iii)* these median forecasts and contemporaneous time-series information is used by the MHW program to update the forecasts and prediction interval bounds. Step *(i)* also decompose the time series (TS) into level, trend (rate of change), seasonal, and approximately serially uncorrelated residual TS components.

Using longitudinal data from 01/1996 to 06/2004 the authors find that the MHW produces reasonably accurate median 2- and 3-month horizon forecasts for the considered non-stationary time-series, *i.e.*, 92% of the 24 time-series forecasts generated (2 forecast horizons, 3 diseases, and 4 age categories = 24 time-series forecasts) have mean absolute percentage errors about 25%. In their experiments the Mean Absolute Percentage Error (MAPE) is smaller for the forecasts of monthly consultation rates for malaria and ARI, while the accuracy decreases for diarrhea’s consultation rates. 

Other time series approaches have been used to explore the issue of extreme climatic events’ impacts. [20] perform time series analyses to estimate the temperature-mortality association for eleven eastern U.S. cities from 1973 to 1994. By using log-linear models for time series data the authors find the following evidence: *(i)* current and recent days’ temperature are the weather factor most strongly predictive of mortality; *(ii)* a threshold temperature appears to exist below which mortality tends to decrease as temperature increases from the coldest days, and above which mortality risk increases as temperature increases; *(iii)* a strong association exists between mortality associated to extreme temperatures and latitude.

[21] use time-series models to analyze mortality due to thermal stresses during heat waves compared to total mortality occurring throughout the whole summer, to understand what fraction of the total impact is attributable to temperature extremes. In the same context, [22] estimate the heat-related mortality due to climate change in Europe, using time-series data and taking into account the threshold temperature where mortality is lowest. The findings suggest that European population have adapted to average summer temperatures, and might adapt to future higher temperatures with only a minor increase in heat-related deaths. These studies suggest that the process of acclimatization should be taken into account when assessing the impact of heat waves and increased temperatures.

Finally we mention [23], who present a time-series analysis of the relationship between El Niño/Southern Oscillation (ENSO) and the prevalence of cholera in Bangladesh using mortality data recorded on a monthly period from 1893 to 1940. Singular spectrum analysis (SSA) is used to capture discontinuous dynamics and trends. The technique allows to decompose the irregular dynamics of the time series and to isolate the inter-annual variability of the data. Their findings suggest that ENSO is responsible for more than 70% of the dynamics of the disease, this relationship being discontinuous in time. In the context of cholera, other time series studies include [24,25,26,27,28]. In particular it is worth mentioning the study by [26], who propose a threshold model, applied to water-borne diseases and cholera, to treat the transition between different states in a more gradual way and to capture the change in the variance of disease.

### 2.2. Panel Data and Spatial Models

#### 2.2.1. Methods

Many economic, socio-demographic, environmental and climatic variables are observed through time (*t* = 1,...,*T*) and across “individuals” (*i* = 1,...,*N*), where the notion of “individual” used in the present context is broad enough to embrace real individuals, households, countries, geographical areas, firms, economic sectors, *etc*. A variable observed through time and across individuals, *Y_it_*, is said to have a panel data structure [29].

Modern econometrics and statistics distinguish between two broad classes of static models for panel data, fixed effect and random effects models. Although both approaches share the same idea of taking into account one major feature of panel data, namely individual heterogeneity, they provide radically different ways of modelling individual variability. The fixed effects model assumes that individual heterogeneity can be represented via individual-specific constants, as:


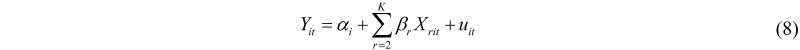


where *u_it_* is a classical error term. This model is appropriate if individual heterogeneity is systematically distributed among individuals, *i.e.*, the sample of data is non-random. Since individual heterogeneity is represented by the additional regressors *α_i_*, correlation between explanatory variables *X_it_* and individual heterogeneity is allowed for in the fixed effects model. On the contrary, the random effects model assumes that individual heterogeneity is randomly distributed among individuals, hence it has to be represented as a classical random normal variable *µ_i_*, which contributes to a composite error term, *v_it_*:


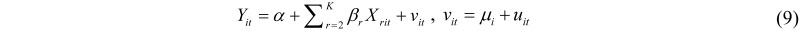


OLS is consistent for the parameters *β_r_*, *r* = 2,…,*K*, of model (8), while GLS is consistent for the parameters in model (9). Since individual heterogeneity is part of the model error term in equation (9), correlation between individual heterogeneity and the explanatory variables *X_it_* would lead to inconsistent estimates.

In applied statistics the autocorrelated structure of many time series variables is widely acknowledged. The simplest way to allow for data autocorrelation is to extend model (9) to include the lagged dependent variable as an additional regressor (dynamic panel data models). Unfortunately, the lagged dependent variable is correlated with the composite error term *v_it_*, leading to inconsistency of the LS estimators. This inconsistency is still present if the variables involved in model (9) are transformed in first differences, in order to eliminate the random effects *μ_i_*:


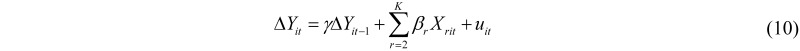


Equation (10) is typically estimated with instrumental variables techniques (e.g., Anderson-Hsiao and Arellano-Bond estimators).

When sample data have a natural location component, two problems arise, namely spatial heterogeneity and spatial dependence (see [30]; for an introduction to spatial econometric models see, among others, [31,32]). Spatial heterogeneity (SH) refers to the fact that many phenomena lead to structural instability over space, in the form of different response functions or systematically varying parameters. SH induces familiar problems such as heteroskedastic random coefficient variation and switching regressions. Spatial dependence (SD) occurs when sample data observations exhibit correlation with reference to points or location in space. Formally, one observation associated with a location *i* depends on other observations at locations *j*, *j ≠ i*, that is *Y_i_ = f (Y_j_)*, *i* = 1,…,*N*; *j* ≠ *i*. In general, the dependence is among several observations, as the index *i* can take on any value from *i* *=* 1,*...*,*N*.

Two reasons are commonly given to explain SD. First, data collection of observations associated with spatial units might reflect measurement errors. Second, the spatial dimension of socio-demographic, economic or regional activities (e.g., environment and climatic variables) may be an important aspect of a modelling problem.

In spatial data analysis the spatial structure of the observations is made explicit by means of spatial weight matrices. The elements of the weight matrix are non-stochastic and exogenous to the model and derived from alternative criteria, such as contiguity (neighbouring units should exhibit a higher degree of spatial dependence than units located far apart), Cartesian space (physical distance matters), non-geographic factors (economic/social proximity).

The presence of spatial correlation between the units of observations can be detected by means of tests which capture the extent to which similarity in values matches with similarity in locations. In this context, positive spatial correlation exists if likewise values tend to cluster in space; negative correlation exists if the locations are surrounded by neighbour with dissimilar values; zero spatial correlation implies that it is not possible to identify a specific spatial pattern of values. This situation is also described as spatial randomness, as values observed at a location do not depend on values observed at neighbouring locations.

#### 2.2.2. Examples

A subject that is contiguous but relevant for the impact of climate on health and its ethical implications is the relationship between pollution and income. The authors of [33] used and extended spatial econometric analysis to investigate whether an inverse-U relationship exists between various pollution indicators and county *per capita* GDP in the U.S. (the so-called environmental Kuznets curve, EKC). The authors emphasize that the EKC is conditional on various structural features (e.g., technology, education, political practices) of each locality. Moreover, they expand the analysis including ethnic diversity among the covariates and by controlling for spatial dependence. Their initial results support the existence of the EKC relationship. The inclusion of spatial autocorrelation is found to raise the turning point of the curve. Another result is that more ethical diverse counties are more polluted. Finally, incorporating a cubic term for income, they find that the toxicity index eventually increases again as income continues to rise.

In [34] the patterns of diseases and mortality rates are analyzed in the framework of the literature on epidemiologic transition, as defined by [35]. The authors provide a cause-of-death analysis for WHO data on mortality by age and sex and recorded cause from 1950 to 2002, and use models for compositional data. Specific causes of death are modeled as a function of the overall level of mortality and the income *per capita*. The findings suggest that considerable variations in cause-of-death patterns across countries and over time are coupled with empirical regularities. Indeed, as mortality levels decline the composition of the causes changes. The effects of mortality declines are more noticeable for children and young adults (with a shift from Group 1 diseases—infectious and parasitic diseases, respiratory infections, maternal conditions, *etc*.—to Group 2 diseases—diabetes, endocrine disorders, *etc*.—and Group 3—injuries—in proportions that vary according to age and sex). In older adults, the composition of mortality remains stable while deaths shift to older ages. Moreover, in many societies, “protracted and polarized” epidemiologic transitions reflect heterogeneity of the social strata. 

### 2.3. Non-Statistical Approaches

#### 2.3.1. General Equilibrium Models: Methods and Examples

A CGE model is composed by a system of linear and non-linear equations which describe consumers’ and producers’ behaviors which are explicitly derived as optimal solutions of utility maximization and cost minimization problems. The system of equations is numerically solved to simulate market equilibrium (see, among others, [36]). CGE models have been used to estimate the welfare costs (or benefits) of health impacts of climate variables. 

The authors of [37] conduct first a meta-analysis of aggregated effects of a change in temperature on mortality for total, cardiovascular and respiratory mortality. Second, he combines these effects with projections of changes in baseline climate conditions of 20 cities, according to climate change scenarios of three General Circulation Models (GCMs). The author finds that for most of the cities included, global climate change is likely to lead to a reduction in mortality rates due to decreasing winter mortality. This effect is most pronounced for cardiovascular mortality in elderly people in cities which experience temperate or cold climates at present.

Similar to [37], [38] considers GCM (General Circulation Models) based studies’ results to estimate (and evaluate in monetary terms) the impacts of climate change for a wide range of market and non-market sectors (agriculture, forestry, water, energy, costal zones and ecosystems, as well as mortality due to vector-borne diseases, heat stress and cold stress). The author estimates that small increases in temperatures would bring some benefits (mainly for the developed world). The conclusion on the global impact of climate change depends crucially on the weights used to aggregate the regional values. Using the simple sum the benefits amount to 2% of GDP. Considering globally averaged prices to value non-markets goods the impact is a 3% reduction of global income. According to equity (ratio of global to regional *per capita* income) -weighted results the world impact is null. Global impacts become negative beyond 1 °C increase in temperatures.

In [39] its authors make use of the General Equilibrium Model (GTAP) in an unconventional approach in order to analyze how health impacts would affect the general economy. Their aim is to estimate the indirect costs on the economic system derived from the health effects as a result of an increase of one degree Celsius in global mean temperature. They estimate the impact on labor productivity and health care expenditures for both the public system and private households, as well as the impacts on GDP. Six health outcomes are considered (cardiovascular disease, respiratory disease, diarrhea, malaria, dengue and schistosomiasis). The impacts on health are taken from different studies (see, for instance, [38,40,41]) that estimate the change in mortality due to an increase of one degree in the global mean temperature. Using data of GTAP model of [42,43] (see the paper and the references therein for a more accurate description) the authors find an increase in mortality and morbidity due to respiratory illness, malaria, dengue fever and diarrhea, with increased costs of illness. In contrast, they evidence a decrease in cardiovascular diseases and schistosomiasis, which dominate the overall impact, leading to a negative trend in the additional expenditure for health care in all countries.

Although the results of [39] go on the same direction (but with stronger evidence) as the conclusions of earlier papers (e.g., [37] and [38]), they are controversial. Indeed, [44] challenges [38,39,45]. The authors argue that the results obtained by [39] are biased due to the omission of extreme weather events and human adaptation to gradual temperatures changes. The main concern is about the use of average temperatures instead of increased variability in local temperatures, which results in an increase of the frequency of extreme hot or cold. Another important issue to be considered in this context is related to the population expected to support heat- and cold-related stresses. In [39], as well as in [38], heat stresses are assumed to impact the urban population only, while cold-related diseases are expected to occur in both the rural and urban population. This assumption might have a strong influence on final results and needs therefore to be further analyzed, especially when considering countries with large rural population [46].

As seen above, [39,44] find contrasting evidence, which is partly related to whether or not extreme climatic events are considered. This suggests that what projected changes in temperatures are considered has a big impact on the results. A review of main findings of the economic literature on climate effects is given as a part of the research of [47]. 

#### 2.3.2. Comparative Risk Analyses: Methods and Examples

The comparative risk assessment (CRA) approach has been developed in the late 90s by the WHO with aim of estimating the contribution of that different public health factors make to the global burden of diseases. The CRA is based on the following data for each risk factor: *(i)* the current and predicted risk distribution of the risk factor; *(ii)* the exposure-response relationship of the associated disease; *(iii)* the total burden of diseases (e.g., DALYs) lost to the various diseases associated with the risk factor. The proportion of the total burden of a disease that is attributable to e specific risk factor is called Impact Fraction and is defined as:


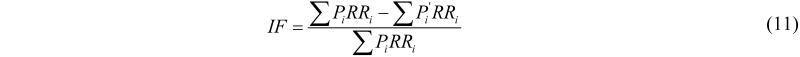


where 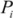 is the proportion of population in the exposure category, 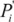 is an alternative proportion and 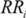 is the relative risk exposure at category *i* compared to the reference level.

Using CRA, which integrate climate models and the evaluation of the health effects of rising temperatures, [48] estimate the potential gains that would derive from combined preventive measures. The authors provide an estimation of the joint effects of 20 selected leading risk factors in 14 epidemiological sub-regions (as a proxy of the world). Among the major risk factors they include environmental risks (such as unsafe water, sanitation and hygiene) that are correlated with the climate. As a tool for the estimation they define the potential impact factor (PIF) as the reduction in population diseases burden or mortality that would occur if the current exposures to multiple risk factors were reduced to an alternative exposures distribution (see the article for a formal definition of the PIF). They find that globally 47% of premature deaths and 39% of total disease burden in 2000 resulted from the joint effects of the considered risk factors. Their results suggest that joint actions would result in a massive reduction of death due to the burden of diseases. Moreover, they find evidence that reducing multiple major risk factors would decrease some of the differences between regions. 

In [2] projections of relative risk attributable to climate change under alternative exposure scenarios using global climate models and CRA are provided. The results are presented for broad WHO geographical regions, and include malaria, diarrhea, malnutrition and heat-related stresses. Referring to malaria, the empirical findings suggest that small temperature increases can significantly affect transmission of the disease. More specifically, temperature increases of 2–3 °C would increase the number of people who are at risk of malaria by around 3–5%. Moreover, the seasonal duration of malaria would increase in many endemic areas. The study presents some limitations which should be investigated in future research in order to estimate the burden of disease. The issue of improved access to water and sanitation systems is not considered, nor is the level of economic development, although these are important factors influencing the population vulnerability. A second limitation is that the correlation between different health outcomes is not evaluated. This is particularly important for malnutrition which is strictly related to occurrence of other diseases. Finally, the model for malaria relates climate variables to geographical areas at risk (and population), instead of disease incidence, and estimates the impacts related to changes in the average temperature while not accounting for climate variability.

The authors of [49] consider the estimates provided by [50] and remark that to generate consistent estimates the models need to incorporate: geographical variation in the vulnerability to climate; future changes in the disease rates due to factors other than climate (e.g., decreases rates of infectious diseases due to technological advances); assumptions on a country’s ability to control a disease such as malaria, dengue fever or diarrheal disease; uncertainties around the exposure-response relationship. Moreover, they claim that, even controlling for the above mentioned (potentially positive or negative) issues, no model can take into account the possibility of irreversibility or plausible low probability events with potentially high impact on human health. As a main consequence, threshold health effects to regulate “tolerable” amount of climate change cannot be identified. Nevertheless, they conclude that more research is needed to reduce the potential impacts of climate change on human health, including the development of improved methods for CRA. 

Finally, [51] relate water-borne diseases with temperature in 14 world regions, showing that the disease incidence tends to increase with temperature. They use multiple regression analysis and include the effect of water supply and sanitation coverage, annual average temperature and *per capita* GDP, taking into account different IPCC climate scenarios. The results show large regional differences in the impacts.

## 3. Integrating Results from Multiple Quantitative Studies: The Case of Malaria

The increases in temperatures may cause changes in the environment with increased risk of malaria transmission. The correlation between the disease and the temperature increase is however complex and multifaceted as it requires a comprehension of how local vectors can spread and transmit. The study of [52] represents a good preliminary discussion of the issue. [53] review a number of studies focusing on the role of climate change in the geographical distributional patterns of malaria. We report here below a review of the main studies existing in this context by classifying them in the three categories of time series, panel data and non-statistical methods.

### 3.1. Time Series Studies

Various time series studies explore the relationship between average temperatures, mid-night temperatures, temperatures in conjunction with rainfall rates, as well as November and December temperatures on malaria. In particular, [54,55,56,57,58] find a significant impact of climate on malaria in Zimbabwe, the Debre Zeit sector of Ethiopia, Rwanda, and the Northwest Frontier Province in Pakistan, respectively. December temperatures coupled with humidity are used by [58] to predict incidence rates of malaria in Pakistan. Other studies consider temperature and deforestation in Tanzania [59] and Kenya [60]. According to the latter study forest clearing has been the cause for increases in malaria transmission. Kenya is considered also by [7]. The main findings of the article are that soil moisture correlates with the human-biting rate of mosquito vectors with a two-week delay. Also soil moisture and entomological inoculation rate are related, with infective parasites taking a six-week time to develop [61].

It has been hypothesized that increasing temperatures could be part of the reason why malaria can now survive at higher altitudes. Many other confounding factors, however, could be causing the increase in malaria in these areas [62]. The dynamics of the geographical spread of malaria are analyzed by [63]. The authors focus on the most important malaria species for humans, *Plasmodium falciparum* and *Plasmodium vivax*, whose range is limited at high altitudes by low temperatures. They investigate whether global warming could drive the geographical spread of the disease and produce an increase in incidence at higher-altitude sites. They use data for four high-altitude sites in East Africa from 1950 to 2006. A nonparametric analysis that decomposes the variability in the data into different components is performed and reveals that the dominant signal in three of the sites and the subdominant signal in the fourth one correspond to a warming trend. To assess the biological significance of this trend, the authors drive a dynamical model for the population dynamics of the mosquito vector with the temperature time series and the corresponding detrended versions. This approach suggests that the observed temperature changes would be significantly amplified by the mosquito population dynamics with a difference in the biological response at least one order of magnitude larger than that in the environmental variable. By using parametric models they also find the existence of significant (linear) trends.

In [64] whether the reemergence of malaria in Western Kenya could be attributed to changes in meteorological conditions is investigated. The existence of trends in a continuous 30-year monthly malaria incidence dataset (1966–1995) is tested for. Malaria incidence increased significantly during the 1966–1995 period. In contrast, no aspect of climate is found to have changed significantly-neither the temperature extremes (maximum and minimum) nor the periods when meteorological data were transformed into months when malaria transmission is possible. Therefore, the authors conclude that climate changes have not caused the highland malaria resurgence in Western Kenya. They suggest that two other factors may have influenced the increase in malaria hospitalizations: an increase in malaria severity indicated by an increased case-fatality rate (from 1.3% in the 1960s to 6% in the 1990s) that is most likely linked to chloroquine resistance. Secondly, travel to and from the Lake Victoria region by a minority of the tea estate workers also exerts an upward influence on malaria transmission in Kericho, Kenya, since such travel increases the numbers of workers asymptomatically carrying gametocytes, which infect. 

### 3.2. Cross-Section and Panel Data Studies

The spatial variation of malaria is analyzed by [65], who examine malaria-related hospital admissions and in-hospital mortalities among children in Africa. The authors apply spatial regression models to quantify the spatial variation of the two outcomes. Using pediatric ward register data from Zomba district, Malawi, between 2002 and 2003, as a case study, they develop two spatial models. The first is a Poisson model applied to analyze hospitalization and minimum mortality rates, with age and sex as covariates. The second is a logistic model applied to individual level data to analyze case-fatality rate, adjusting for individual covariates. The results show that rates of hospital admission and in-hospital mortality decrease with age. Case fatality rate is associated with distance from the hospital, age, wet season, and increases if the patient is referred to the hospital from the primary health facilities. Furthermore, death rates are high on the first day, followed by relatively low rates as the length of hospital stay increases. The outcomes show substantial spatial heterogeneity, which may be attributable to the varying determinants of malaria risk, health services availability and accessibility, and health seeking behavior. Moreover, the increased risk of mortality of referred children may imply inadequate care being available. The authors suggest that reducing the burden of malaria requires integrated strategies that encompass availability of adequate care at primary facilities, introduce home or community case management and encouraging early referral. Those interventions would be needed to interrupt malaria transmission.

In a subsequent article [66], the author extends the analysis of [65] to profile spatial variation of malaria risk and analyze possible association of disease risk with environmental factors at sub-district level in northern Malawi. Using the same data on malaria incidence the author compares Bayesian Poisson regression models assuming different spatial structures. For each model he adjusts for environmental covariates initially identified through bivariate non-spatial models. The model with both spatially structured and unstructured heterogeneity is shown to provide the best fit, based on models comparison criteria. Malaria incidence appears to be associated with altitude (measured in meters above sea level), precipitation (measured in millimeters per annum) and soil water holding capacity. The risk increases with altitude (relative risk (RR): 1.092, with a 95% confidence interval (CI) between 1.020 and 1.169) and precipitation (RR: 1.031, with a 95% CI between 0.950 and 1.120). At medium level of soil water holding capacity relative to low level, the risk is reduced (RR: 0.521, with a 95% CI between 0.298 and 0.912), while at high level of soil water holding capacity relative to low level the risk is raised (RR: 1.649, with a 95% CI between 1.041 and 2.612). Compared to the commonly used standardized incidence ratios, the model-based approach appears to provide homogenous and easy to interpret risk estimates. Generally, the smoothed estimates show less spatial variation in risk, with slightly higher estimates of malaria risk (RR > 1) in low-lying areas mostly situated along the lakeshore regions, in particular in Karonga and Nkhatabay districts, and low risk (RR < 1) in high-lying areas along Nyika plateau and Vwaza highlands. The results suggest that the spatial variation in malaria risk in the region is a combination of various environmental factors, both observed and unobserved. The results also identify what are the areas of increased risk, where further epidemiological investigations could be carried out. 

Another interesting study in this context is the one of [67], who project malaria transmission in new geographical regions in India. According to this study malaria is expected to move from central regions towards South Western and Northern Regions by 2050. Some studies about malaria also project a shift in the duration of transmission windows which might increase or decrease according to the different climatic conditions of a region (see, among others, [67,68]).

The work described in [69] considers the progressive rise in the incidence of malaria over the last decades in African highlands. The phenomenon is largely a consequence of agroforestry development, and is exacerbated by scarce health resources. Moreover, in these areas, where the pattern of malaria is unstable, the epidemic may be precipitated by relative subtle climate changes and therefore requires special monitoring. The authors use mathematical models to identify epidemic-prone regions in highlands Africa, and to quantify the difference expected to occur as a consequence of projected global climate change. To make estimates about the areas that are vulnerable to epidemic outbreaks of malaria, they use data and models from Geographic Information Systems (GIS) (computerized mapping systems) and Remotely Sensed (RS) imagery data from earth-orbiting satellites. Correlations among variables are found. However, the authors observe that since correlation doesn’t imply causality these results are not conclusive and require further investigation. To model the dynamics in highlands malaria in relation to climate change they use an integrated system, scenario-based approach (Integrated Assessment Models; see, among others, [45,47]). Evidence is found that the direct influence of climate may contribute to malaria risk. However, this effect cannot be claimed to be the most important determinant of malaria transmission. The effects of temperature on mosquito development, feeding frequency, longevity and incubation period are estimated. The model is linked to baseline climatology data from 1931 to 1960 and uses integrated techniques to generate climate scenarios. Their findings suggest that is not possible to prove that any single factor has caused the outbreaks in African highland. Projected climate changes are likely to modify the epidemics in the regions: 260–320 million more people are projected to be affected by malaria by 2080 as a consequence of new transmission zones [70].

### 3.3. Non-Statistical Studies: General Equilibrium

The authors of [37] propose a system-oriented analysis, based on scenarios of projected temperature, and that considers joint effects (rather than phenomena in isolation) to assess the future impacts of climate change. In his analysis he examines the effects of climate change on vector-borne diseases, on thermal-related mortality, and the effects of increasing ultra-violet levels due to ozone depletion on skin cancer. Considering malaria the author defines the basic reproduction rate in an area (R_0_) as the vector capacity multiplied by the duration of the infectious period in humans. The factors that are involved in the calculation of (R_0_) include: the mosquitoes/people ratio, the number of mosquito bites per person per day, the probability that an infected mosquito infects a human, the chances that a mosquito becomes infected during a blood meal, the incubation period, and the daily survival probability of the mosquito. Indirect factors include: the availability of breeding sites which is related to precipitation, human population density, human population migration, the feeding habits of the mosquitoes, the presence of other animals on which the mosquitoes feed, human exposure (which can be affected by the use of bed nets or other interventions), temperature the immunological and nutritional status of the population, the effectiveness of medical treatment, natural enemies of the mosquitoes, and control efforts. This model is further complicated by algorithms that predict changing genetic adaptations in the parasite and vector that lead to resistance. Based on this approach, evidence is found that the number of people in developing countries likely to be at risk of malaria infection will increase by 5–15% because of climate change, depending on which the Global Circulation Model (GCM) and climate change scenario is used. The areas that are expected to have the most increase in malaria transmission are ones at the fringes of transmission. Unless they are able to use effective control strategies, these regions have low levels of immunity and are likely to experience epidemics [37].

In general, there is considerable uncertainty about the magnitude of the overall impact of malaria. While some models project a net increase in the population exposed to malaria (and in the incidence rate) due to climate change [82], others have found only minor changes in malaria distribution [2]. This uncertainty is due to the complex dynamics underlying the transmission of this vector and to other important factors such as the socio-demographic and environmental factors which are playing a substantial role in the transmission mechanism.

## 4. Using Quantitative Results Toward Managing Human Health

The previous section has concentrated on recent quantitative contributions on the relationship between climate and health. However, since this issue involves different disciplines and approaches, a broader view becomes necessary, in order to provide a better understanding of the interactions between climate and health. In this section we present a critical summary of the recent literature related to the following more general aspects of the impacts of climate changes on human health: the economics of climate change; how to manage the health effects of climate change; Early Warning Systems and infectious diseases. 

### 4.1. The Economics of Climate Change

Reference [47] is a key reference giving a complete framework of the economics of climate change. The book reviews scientific and geological basis of the studies on climate change’s impacts. For example, it lists the possible impacts associated to 1, 2 up to 5 °C of temperature increase. Restricting to the effects for health, these include a larger (and increasing exponentially with temperatures) number of deaths caused by diseases such as malaria, diarrhea and malnutrition at lower latitudes (Africa); and a reduction in winter deaths at higher latitudes (Northern Europe, USA). The author considers the ethical implications of the disproportionate distributions of impacts across regions and populations, and provides a series of policy recommendations. For the problem at stake, the chapter that concerns the economic analyses of climate change costs is specifically relevant. 

The measurement of costs of climate (measured on income/consumption, health and environment dimensions) is a challenging task. The main reasons being that this kind of analyses involves the use of variables and projections that are highly uncertain (however, according to the author, omitting some of uncertain but potentially most damaging impacts has caused some early attempts to underestimate the costs of climate change). Moreover, the effects can be seen only over several decades and with a long-time delay. Based on a review of the studies of the costs of climate warming, the author concludes that the Integrated Assessment Models (IAM) constitute a valid methodological foundation; however first-round IAM studies consider the effects of climate at temperatures that are now likely to be exceeded. The mixed evidence found by different authors crucially relies on what increase in temperature is considered. Indeed, there is a common evidence that the warming above 3–4 °C would reduce global welfare, and that and temperatures increases of 5–6 °C are estimated to result in a 5%–10% reduction in global GDP relative to the “no-climate-change” scenario.

In the methodological framework of IAM, [47] estimates the BAU (business as usual) costs of climate: he estimates the costs to be equivalent to a *per capita* reduction of income of 5% at a minimum. This proportion could increase to 11% by considering the direct effects on environment and health (“non-market” impacts [83]). In case it turns out to be true that the responsiveness of climate system to greenhouse gas emissions is larger than what previously thought, the costs would increase even more. Finally there is a noticeable disproportion in the distribution of the burden of climate change impact among developing and rich countries. As regards health, the major impacts are in Sub-Saharan Africa and Asia, which are already facing a considerable burden of disease. Developing countries are actually tackling with more constraints. On the one hand they are expected to face high population growth with increased risk of poor housing, hunger and infectious diseases due to poor water and sanitation systems. On the other hand, their adaptive capacity is limited in terms of financial and infrastructural resources, health care system, poor health status of the population and poor capacity of collecting and analyzing data. Additional problems are related to income inequalities, migration and conflicts. As stated in [84], priorities for research should include the development of methods to provide more quantitative assessments of climate change impacts in low- and middle-income countries.

### 4.2. Managing the Health Effects of Climate Change

Global warming is expected to increasingly impact food security, water availability and quality, and exact a toll on public health, spurring chronic disease, malaria prevalence, and cardiovascular and respiratory diseases.

Current weather conditions heavily impact the health of poor people in developing nations, and climate change has a multiplying effect. A changing climate further affects the essential ingredients of maintaining good health: clean air and water, sufficient food and adequate shelter. A warmer and more variable climate leads to higher levels of some air pollutants and increases transmission of diseases through unclean water and contaminated food. It compromises agricultural production in some of the least developed countries, and it increases the hazards of weather-related disasters.

Therefore global warming, together with the changes in food and water supplies it causes, can indirectly spurs increases in such diseases as malnutrition, diarrhea, cardiovascular and respiratory diseases, and water borne and insect-transmitted diseases. This is especially worrisome because a massive number of people are already impacted by these diseases. Also, there is an inter-relationship among these health outcomes. For example malnutrition is linked with malaria and diarrhea which can cause significant weight loss in affected children when accompanied with food scarcity. Malaria and diarrhea can be both cause and effect of malnutrition.

“Managing the Health Effects of Climate Change” is a wide multidisciplinary overview of the major threats - both direct and indirect - to global health from climate change, carried out by [85]. Effects of predicted climate change are described by the authors and actions to be undertaken are discussed. 

The starting point of the analysis is that during this century, the Earth’s average surface temperature rises are likely to exceed the safe threshold of 2 °C above preindustrial average temperatures. Rises will be greater at higher latitudes, with medium-risk scenarios predicting 2–3 °C rises on average by 2090 and 4–5 °C rises in northern Canada, Greenland, and Siberia.

Health effects of the predicted climate change will cause vector-borne diseases to expand their reach and death tolls, especially among elderly people. Moreover, the indirect effects of climate change on water, food security, and extreme climatic events are likely to have even bigger effects on global health.

An integrated and multidisciplinary approach to reduce the adverse health effects of climate change requires at least three levels of action. First, policies must be adopted to reduce carbon emissions and to increase carbon biosequestration, and thereby slow down global warming and eventually stabilize temperatures. Second, further research is needed to understand clearly the links between climate change and disease occurrence. Third, appropriate public health systems should be put into place to deal with adverse outcomes in terms of efficient and cost-effective adaptation measures at local, and national levels.

Reference [85] considers what the main obstacles to effective adaptation might be, focusing on six aspects that connect climate change to adverse health outcomes: changing patterns of disease and mortality, food, water and sanitation, shelter and human settlements, extreme events, and population and migration. Each is considered in relation to five key challenges to form a policy response framework: informational, poverty and equity-related, technological, sociopolitical, and institutional.

Our capacity to respond to the negative health effects of climate change relies on the generation of reliable, relevant, and up-to-date information. Strengthening informational, technological, and scientific capacity within developing countries is crucial for the success of a new public health movement. This capacity building will help to keep vulnerability to a minimum and build resilience in local, regional, and national infrastructures.

Few comprehensive assessments on the effect of climate change on health have been completed in low-income and middle-income countries, and none in Africa. The report endorses the [86] recommendations for full documentation of the risks to health and differences in vulnerability within and between populations; development of health protection strategies; identification of health co-benefits of actions to reduce greenhouse gas emissions; development of ways to support decisions and systems to predict the effect of climate change; and estimation of the financial costs of action and inaction. Policy responses to the public health implications of climate change will have to be formulated in conditions of uncertainty, which will exist about the scale and timing of the effects, as well as their nature, location, and intensity.

A key challenge is to improve surveillance and primary health information systems in the poorest countries, and to share the knowledge and adaptation strategies of local communities on a wide scale. Essential data need to include region-specific projections of changes in health-related exposures, projections of health outcomes under different future emissions and adaptation scenarios, crop yields, food prices, measures of household food security, local hydrological and climate data, estimates of the vulnerability of human settlements (e.g., in urban slums or communities close to coastal areas), risk factors, and response options for extreme climatic events, vulnerability to migration as a result of sea-level changes or storms, and key health, nutrition, and demographic indicators by country and locality.

In the view of the commission the key factors to management of health effects of climate change will be: reduction of poverty and inequity in health; incentives for the development of new technologies and application of existing technologies in developing countries; change in lifestyle; improved coordination and accountability of global governance; increase advocacy to reduce climate change trough public health awareness.

### 4.3. Developing Diseases and Early Warning Systems

Considerable research is currently being conducted to elucidate linkages between climate and epidemics. Of the 14 diseases meeting the defined criteria for potential for climate-informed Early Warning Systems EWS, few (African trypanosomiasis, leishmaniasis and yellow fever) are not associated with some sort of EWS research or development activity. For the West Nile virus, an operational and effective warning system has been developed which relies solely on detection of viral activity and it remains unclear whether the addition of climatic predictors would improve the predictive accuracy or lead-time. For the remaining diseases (cholera, malaria, meningitis, dengue, Japanese encephalitis, St Louis encephalitis, Rift Valley Fever, Murray Valley encephalitis, Ross River virus and influenza), research projects have demonstrated a temporal link between climatic factors and variations in disease rates. In some of these cases the power of climatic predictors to predict epidemics has been tested. 

The research reviewed in this report demonstrates that climate information can be used to improve epidemic prediction, and therefore has the potential to improve disease control. In order to make full use of this resource, however, it is necessary to carry out further operational development. The true value of climate-based early warning systems will come when they are fully integrated as one component in well-supported systems for infectious disease surveillance and response. 

## 5. Conclusions

This paper has focused on the critical evaluation of recent quantitative assessments of health risks associated with climate change. The main contribution of our paper is to offer an integrated vision of the main scientific conclusions on the effects of climate change on human health, which are supported by the use of formal qualitative analyses.

In this respect, the journal articles surveyed in this paper have been classified according to the quantitative models adopted, which have been identified in the broad classes of time-series models, cross-section, panel and spatial models, and non-statistical approaches, such as computable general equilibrium models and comparative risk assessments. Moreover, our paper has presented a critical summary of the recent literature related to more general aspects of the impacts of climate changes on human health, such as: the economics of climate change; how to manage the health effects of climate change; Early Warning Systems and infectious diseases; evaluating the risks to human health which are related to climate change; the implications of modifiable environmental risk factors. 

Climate change is already affecting human health, livelihoods, safety, and society and the expectation is that these effects will become greater. The climate impact is still difficult to assess with accuracy because it results from a complex interplay of factors. It is challenging to isolate the human impact of climate change definitively from other factors such as natural variability, population growth, land use and governance. In several areas, the base of scientific evidence is still not sufficient to make definitive estimates with great precision on the human impacts of climate change. However, data and models do exist which form a robust starting point for making estimates and projections that can inform public debate, policy-making and future research. 

The pressure for increased precision in estimates presents a rallying cry for investment in research on the social implications of climate change. Three areas which require additional research in the near future are:

– The attribution of weather-related disasters to climate change, as no consensus estimate of the global attribution has yet been made;
– Estimate of economic losses today, as the current models are forward looking;
– Regional analysis, as the understanding of the human impact at regional level is often very limited but also crucial to guide effective adaptation interventions.
